# Primary malignant melanoma of the cervix: Report of 14 cases and review of literature

**DOI:** 10.18632/oncotarget.17183

**Published:** 2017-04-18

**Authors:** Guangwen Yuan, Lingying Wu, Bin Li, Jusheng An

**Affiliations:** ^1^ Department of Gynecologic Oncology, National Cancer Center/Cancer Hospital, Chinese Academy of Medical Sciences, Peking Union Medical College, Beijing, 100021, China

**Keywords:** malignant melanoma, treatment, cervix, prognosis

## Abstract

**Purpose:**

To investigate the clinical characteristics and prognosis of primary malignant melanoma of the uterine cervix.

**Results:**

The median age of the patients was 61.2 years (range, 42-78 years). The median overall survival of the patients at stage I, II and III were 39.2 months, 47.8 months and 9.0 months (P=0.574) and the 2-year overall survival for each stage were 80.0%, 50.0% and 0.0% respectively. Twelve (85.7%) patients developed recurrence and eleven patients (78.6%) died. Ten patients received surgery and four patients were treated with chemotherapy and/or radiotherapy. Immunotherapy was administrated to two patients after surgery. The mean survival time of patients with surgery and without surgery were 47.9 vs.7.75 months (P=0.047). Patients received radical hysterectomy had longer survival than patients underwent total hysterectomy (66.8 months vs 19.5 months, P=0.016).

**Methods:**

Clinical data from 14 patients with primary malignant melanoma of the cervix between January 1981 and December 2014 were reviewed.

**Conclusions:**

Patients with primary malignant melanoma of the cervix have a poor prognosis. Radical hysterectomy and pelvic lymphnode dissection may offer better prognosis for stage I and II patients.

## INTRODUCTION

Malignant melanoma (MM) is a rare malignant neoplasm of the skin and mucous membranes and accounts for about 1% of all cancers [1]. The mucosal malignant melanomas account for 0.03% of all cancers and occur in a variety of sites including the oral cavity, esophagus, anus, conjunctiva and gynecologic tract [2]. 5% female MM occur on the vulva, with rare cases detected in the ovary, uterus and cervix [3].

Current treatment for mucosal melanoma is primarily based on experience from cutaneous mela-noma. Optimal management of cervical MM have not achieved consensus due to scarcity [4–7]. To our knowledge a maximal cohort of 83 patients with primary MM of cervix were reported, and exhibited poor long term survival [8].

Here we reported 14 cases of primary MM of cervix and reviewed the literature. Our aim was to explore the best treatment strategy for this rare disease.

## RESULTS

### Patient characteristics

Fourteen patients with primary MM of cervix were included in our study. The mean age was 61.2 years. All patients presented with vaginal bleeding. The maximum tumor diameter ranged from 2.0cm to 7.0cm. The tumor was exclusively or primarily located in cervix regardless of vaginal involvement. There was no sign of vulval lesion. Patients` final stages were 5 stage I, 6 stage II and 3 stage III. Main clinicopathologic characteristics are summarized in Table 1.

**Table 1:** Characteristics of 14 patients with primary malignant melanoma of the cervix

No.	Age (years)	BMI	Presenting symptom	Comorbidities	The largestdiameter oftumor (cm)	FIGO Stage
1	61	25.8	vaginal bleeding	Hyperthyreosis	3.5	IIB
2,	74	22.6	vaginal bleeding	Hypertension	6	IB2
3	56	25.3	vaginal bleeding	None	4.5	IIIB
4	74	27.4	vaginal bleeding	None	3	IIA1
5	77	21.0	vaginal bleeding	Tricuspid incompetence	2	IB1
6	45	26.5	vaginal bleeding	None	2	IB1
7	50	32.4	vaginal bleeding	None	7	IB2
8	58	26.3	vaginal bleeding	None	2	IB1
9	57	28.1	vaginal bleeding	arrhythmia	4	IIA1
10	42	30.8	vaginal bleeding	None	7	IIB
11	63	26.3	vaginal bleeding	None	5	IIIB
12	54	29.7	vaginal bleeding	none	6	IIB
13	78	23.8	vaginal bleeding	Pulmonary emphysema	2	IIIB
14	68	20.0	vaginal bleeding	None	2	IIA1

FIGO: International Federation of Gynecology and Obstetrics

### Treatments

The detailed treatment approaches are presented in Table 2. A total of ten patients underwent surgery, six with radical hysterectomy (RH) and four with total hysterectomy (TH). Pelvic lymph node dissection was omitted in ** patients received TH. Stage IIB patient No.10 received preoperative radiotherapy. Patient No.12 with stage IIB had preoperative radiotherapy and chemotherapy. Post-surgery chemotherapy (dacarbazine and cisplatin) and radiotherapy were performed in five and two cases respectively. Two patients were administrated biological therapy (interleukin-2 and interferon alfa) after surgery. Patients No.5, No.6 and No.14 did not receive any therapy following surgery.

**Table 2:** Treatment methods and survival

No	Stage	NC	POR	Surgery	PS-C	PS-R	PS-B	R or P	Status	OS(months)
1	IIB	-	-	RH+PLND	-	-	Y	-	Alive	193.0
2	IB2	-	-	RH+PLND	Y	-	-	R	Dead	33.0
3	IIIB	-	-	No surgery	Y	Y	-	P	Dead	5.0
4	IIA1	-	-	TH+PLND	Y	-	Y	R	Dead	28.0
5	IB1	-	-	TH	-	-	-	R	Dead	25.0
6	IB1	-	-	RH+PLND	-	-	-	-	Alive	87.0
7	IB2	-	-	TH+PLND	Y	-	-	R	Dead	16.0
8	IB1	-	-	RH+PLND	Y	-	-	R	Alive	35.0
9	IIA1	-	-	No surgery	Y	-	-	P	Dead	4.0
10	IIB	-	Y	TH	-	Y	-	R	Dead	9.0
11	IIIB	-	-	No surgery	Y	Y	-	P	Dead	10.0
12	IIB	Y	Y	RH+PLND	Y	Y	-	R	Dead	33.0
13	IIIB	-	-	No surgery	Y	Y	-	P	Dead	12.0
14	IIA1	-	-	RH+PLND	-	-	-	R	Dead	20.0

RH: radical hysterectomy

TH: total hysterectomyNC: Neoadjuvant ChemotherapyPOR: Preoperative Radiotherapy

PS-C: Post-surgery chemotherapy

PS-R: Post-surgery radiotherapy

PS-B: Post-surgery biological therapy

R: Recurrence

P: Persistence

Y: received the treatment

-: did not receive the treatment

Three patients only received chemotherapy and radiotherapy due to advanced stage. Patient No.9had surgical contraindication (arrhythmia)received chemotherapy.

### Recurrences and survival

The median follow-up time was 22.5 months (4.0 to 193.0 months). Twelve (85.7%) patients developed recurrences or presented persistent disease. Eleven (78.6%) died of the disease. All the four patients dismissed surgery had persistent disease. Time interval for recurrence from first treatment varied between 2.0 to 24.0 months post (Table 3).

**Table 3:** Treatment methods and survival post recurrence

No	Stage	PFI(months)	Recurrent site	Treatment P-R	OS P-R(months)	Status	OS(months)
2	IB2	24.0	skin of back	N	6.0	Dead	33.0
4	IIA1	12.0	Vagina	C	13.0	Dead	28.0
5	IB1	12.0	vagina	S+C	13.0	Dead	25.0
7	IB2	2.0	vagina	S+C	11.0	Dead	16.0
8	IB1	10.0	vagina	S+C	25.0	Alive	35.0
10	IIB	3.5	skin of lower limb	N	3.5	Dead	9.0
12	IIB	11.5	skin of lower limb	N	7.5	Dead	33.0
14	IIA1	4.5	skin of upper limb	S	15.5	Dead	20.0

PFI: progression free interval

P-R: Post recurrence

OS: overall survival

S: surgery

C: chemotherapy

N: no treatment

Four patients relapsed in vagina. Four patients had limb or back recurrence. Three patients received surgery and chemotherapy for recurrence;10, 13 and 25months survival were observed. 15.5 and 13.0 months of after-recurrence survival were recorded for two patients underwent surgery and chemotherapy respectively. Three patients did not receive any treatment after recurrence because poor status or economic reason, and lived 3.5 months, 6.0 months and 7.5 months.

Two patients (No. 1 and No. 6) remained without evidence of disease; one patient (No. 8) was alive with disease at the time of last follow-up. Two patients (14.3%, 2/14) lived for more than five years and seven (50.0%, 7/14) survived over two years (Table 2).

The mean survival time of patients with or without surgery were 47.9 and 7.75 months respectively (P=0.047). The patients who did not receive surgery had persistent disease and died at 4, 5, 10 and 12 months.

Of the 10 patients who underwent surgery, eight patients developed recurrences and 7 patents died of it. The mean survival time of four patients received TH was 19.5months (9 to 28), and was 66.8 months(20 to 193) for six patients received RH (P=0.016). All patients (3) were alive had radical hysterectomy and pelvic lymph node dissection.

The median OS of the 7 patients underwent surgery and adjuvant therapy was 28.0 months, and six (85.7%, 6/7) of them had recurrence. All three patients without adjuvant therapy experienced recurrence. For them, two died at 25.0 and 20.0 mouths, and one was alive for 51.0 months with residual tumor at the last contact.

The mean OS of the patients at stage I, stage II and stage III were 39.2 months, 47.8 months and 9.0 months respectively(P=0.574). The 2-year survival of each stage were 80.0% (4/5), 50.0% (3/6) and 0.0% (0/3) respectively.

## DISCUSSION

Primary malignant melanomas of cervix is an extremely rare disease which origin from the cervical melanocytic cells [9]. Diagnosis is determined by gynecological examination, histological results and immunohistochemical staining. Before the diagnosis of primary cervical melanoma, we should rule out the existence of melanoma elsewhere. In this study, we adopted the following diagnostic criteria suggested by Morris and Taylor: (i) The presence of melanin in the normal cervical epithelium; (ii) the absence of melanoma anywhere else in the body; (iii) the demonstration of junctional changes in the cervix; and (iv) metastases according to the pattern of cervical carcinoma [10]. This criteria offer the reliable diagnostic results and is adopted by the majority of pathologists and clinicians.

In early stage, the primary MM of the cervix localized to the cervical mucosa, and spreads to vaginal fornix, uterosacral ligaments, vulva and pelvic wall at advanced stages [11, 12]. Lymphatic metastasis usually follows the pattern of the cervical carcinoma. If the vaginal wall is involved, the inguinal lymphnode may be involved [4]. None of ten patients who received surgery at our hospital had lymphnode involvement. By contrast, three out of four patients without surgery had enlarged pelvic lymphnodes.

Current staging system for skin MM is based on tumor thickness and the status of regional lymph nodes [2];by contrast, the clinical presentation and spread pattern of primary MM of cervix is similar to that of cervical carcinoma. The FIGO staging system has been accepted by most researchers [7, 13, 14]. Pusceddu reviewed 78 cases of primary MM of the cervix and found that FIGO stage I, II, III and IV accounted for 41%, 34.4%, 18.0% and 6.5% respectively. We found five (35.7%) stage I, six (42.9%) stage II and three (21.4%) stage III patients in our study. About 80% of the primary MM of the cervix were at the early stages (stage I and stage II). It may be attributed to the obvious symptoms that alert the patients to get a physical examination. Most frequent symptoms of primary MM of the cervix are vaginal bleeding, vaginal discharge, abdominal pain, dyspareunia and post-coital bleeding [15, 16]. The symptom at presentation of all patients in this study was vaginal bleeding.

While little consensus has been achieved about the best management of cervical MM, surgery remains the major therapeutic method [5, 11, 17]. The recommended surgery procedures include hysterectomy, lymph-node dissection and partial or bilateral salpingo-oophorectomyd. Some authors advocated radical hysterectomy to obtain clean surgical margins of at least 2 cm [14, 18]. Other researchers suggested that a more conservative surgical approach may be appropriate considering poor prognosis [6, 17, 19]. In our study, the mean OS of the patients received TH was shorter than patients with RH (19.5months vs 66.8 months, P=0.016). Considering the metastases pattern of primary MM of the cervix is similar to cervical carcinoma [10], RH may improve the prognosis of the primary MM of the cervix. We need more studies to arrive at a conclusion for preferred surgical procedure.

Whether dissection of clinically negative regional lymph-nodes is still controversial. Jones et al found that 30% of patients with clinically normal lymph-nodes at presentation contained microscopic melanoma and suggested regional lymph node dissection [20]. By contrast, Cantuaria and Furuya advocated pelvic and para-aortic lymph node dissection exclusively for cases with grossly enlarged lymph-nodes or with tumor extends beyond the uterus [14, 21]. The value of lymph node dissection for this disease remains arguable [6, 14]. In our study, eight patients received regional lymph node dissection, and no one had lymph node metastasis. No recurrences was detected in lymph nodes. We suggest that regional lymph node dissection should be carried through for patients with primary MM of the cervix particularly for the patients in early stage need more consideration.

Great attempts have been made to evaluate the potential benefits of adjuvant chemotherapy. There is no chemotherapeutic regimen which has valid effect on reducing or delaying the recurrence. Most authors used the chemotherapy for MM of the skin melanoma [4, 5]. Dacarbazine is the most widely used drug with 15-20% response rates [22, 23].

Lpilimumab, a monoclonal antibody directed to the immune checkpoint receptor termed “cytotoxic T lymphocyte antgen-4 (CTLA-4)”, received FDA approval for treatment of metastatic melanoma in March 2011. The approval was based on two randomized phase III trials in which Lpilimumab improved the survival of metastatic melanoma [24, 25]. Recently, some therapies targeted against BRAF mutations (Vemurafenib, Dabrafenib), KIT mutations (Imatinib) and inhibitor of MEK1 and MEK2 (Trametinib) showed good results in advanced stage melanoma [26–29]. These new agents gave us great hope for this kind of disease.

MM is considered a radio-resistant tumor. RT applied as adjuvant, preoperative and palliative treatment in some case reports [5]. Adjuvant pelvic RT might be applied to patients with unsatisfactory surgical resection, infiltrated parametriums and involved lymphnodes [4, 30]. Additionally, primary RT in advanced stage MM of the cervix might shrink tumor [4, 31]. In our study, two patients received post-surgery radiotherapy and died at 9 months and 33 months. Three patients onlyhad radiotherapy and chemotherapy due to advanced stage, and died at 5, 10 and 12 months. Outcomes in patients treated with RT were poorer than those received surgery, reflecting the early stage disease and physical status for surgical cases [17].

Tumor stage is the main prognostic factor of this rare disease [1, 3]. Only a few patients with cervical MM had long-term survival. Pusceddu reported that 10.7% of patients lived more than 5 years. The majority of patients died within 3 years (87.5%) [32]. In our study, 50.0% of patients survived for more than two years, and only two patients (14.3%) survived for more than five years.

The largest case review of primary MM showed the mean and median overall survival were 22.9 and 12 months(range 0.1–168 months)respectively. Mean/median OS stratified by stage were 31.5/20 months (ranges 1–156) for stage I, 22.8 /10.5 (ranges 0–168) for stage II, 14.5 /11 (ranges 6–34) for stage III, and 5.75/5 months (range 1–12) for stage IV [32] in a pervious study. and the corresponding outcomes in our study were39.2 and 33.0 months (range 16-87), 47.8 and 24 months (range 4-193) and 9 and 10 months (range 5-12) respectively (P=0.574).

In conclusion, patients with primary malignant melanoma of the cervix have a poor prognosis and radical hysterectomy may offer better outcome for STAGE I1 OR II2 patients.

## MATERIALS AND METHODS

We reviewed the records of all patients who were presented to the Cancer Hospital of Chinese Academy of Medical Science with a diagnosis of primary malignant melanoma of the cervix from January 1, 1981 to December 31, 2014. Surgical and pathological reports were reviewed. Gynecologic pathologists confirmed all the histopathologic diagnoses. Demographic and clinical information, including age at the time of diagnosis, presenting symptoms, tumor characteristics, treatment and follow-up were obtained from medical records.

Treatment modalities were classified as three categories: surgery, chemotherapy and radiation therapy. Overall survival was the primary end point result.

All data were analyzed by SPSS (version 19.0; IBM Company, the United States). OS was calculated from the date of initial treatment to the date of last follow-up by Kaplan-Meier method. The threshold for significance was set at P<0.05.
